# Protocol based invasive intracranial pressure monitoring in acute liver failure: feasibility, safety and impact on management

**DOI:** 10.1186/s13054-017-1762-6

**Published:** 2017-07-11

**Authors:** Venkatakrishna Rajajee, Robert J. Fontana, Anthony J. Courey, Parag G. Patil

**Affiliations:** 1 Departments of Neurosurgery and Neurology, University of Michigan, 3552 Taubman Health Care Center, 1500 East Medical Center Drive, SPC 5338, Ann Arbor, MI 48109-5338 USA; 2 Division of Gastroenterology, Department of Internal Medicine, University of Michigan, Ann Arbor, MI USA; 3 Division of Pulmonary & Critical Care Medicine, Department of Internal Medicine, University of Michigan, Ann Arbor, MI USA; 4 Department of Neurosurgery, University of Michigan, Ann Arbor, MI USA

**Keywords:** Fulminant hepatic failure, Hepatic encephalopathy, Cerebral edema, Intracranial hypertension, Intracranial hemorrhage

## Abstract

**Background:**

Acute liver failure (ALF) may result in elevated intracranial pressure (ICP). While invasive ICP monitoring (IICPM) may have a role in ALF management, these patients are typically coagulopathic and at risk for intracranial hemorrhage (ICH). Contemporary ICP monitoring techniques and coagulopathy reversal strategies may be associated with a lower risk of hemorrhage. Our objective was to evaluate the safety, feasibility, impact on clinical management and outcomes associated with protocol-directed use of IICPM in ALF.

**Methods:**

Adult patients admitted between June 2011 and October 2016, with ALF and grade-4 encephalopathy with a reasonable likelihood of survival, were eligible for IICPM. The coagulopathy reversal protocol included administration of recombinant Factor VIIa (rFVIIa) and desmopressin, a goal platelet count >50,000/mm^3^ and fibrinogen >100 mg/dL. Monitor insertion was performed within an hour of the rFVIIa dose. Only intraparenchymal monitors were used. Computed tomography of the brain was performed prior to and within 24 hours of monitor placement. Outcomes of interest included ICH, sustained intracranial hypertension, therapeutic intensity level (TIL) for ICP management, mortality and functional outcome on the Glasgow Outcome Scale (GOS) at discharge and 6 months.

**Results:**

A total of 24/37 patients (65%) with ALF underwent IICPM. The most common reason for exclusion was encephalopathy grade <4. Four patients underwent liver transplantation. There was one asymptomatic ICH following IICPM, in a patient who had an excellent outcome. Sustained intracranial hypertension occurred in 13/24 monitored patients (54%), 5/24 (21%) required extreme measures (TIL-4) for ICP control, which were successful in 4 patients: 12/24 patients (50%) died but only 4 deaths (17%) were attributed to intracranial hypertension. Six of the 8 survivors with 6-month follow up had good functional outcome (GOS >3).

**Conclusions:**

Protocol-directed use of IICPM in ALF is feasible, associated with a low incidence of serious complications and has a significant impact on clinical management.

## Background

Acute liver failure (ALF), defined as hepatic encephalopathy and coagulopathy within 26 weeks of illness onset, is a rare but potentially life-threatening illness [1]. Patients with ALF may develop diffuse cerebral edema, thought to result from an elevated serum level of ammonia, which easily crosses the blood-brain barrier then reacts with glutamate to form glutamine within astrocytes [2]. The large quantity of intracellular glutamine is then thought to result in an osmotic shift of fluid into astrocytes and cerebral edema [2]. Elevated cerebral blood flow (hyperemia), possibly as a result of cerebral vasodilatation and impaired autoregulation, may also contribute to cerebral edema and intracranial hypertension [2]. Intracranial hypertension is an important cause of death in patients with ALF [1–7]. Appropriate management of cerebral edema, in conjunction with emergency liver transplantation (LT) in select patients, may result in favorable clinical outcomes [1, 8–20]. Since the bedside physical exam and radiographic imaging are poor predictors of elevated intracranial pressure (ICP) [12, 21], invasive ICP monitoring (IICPM) remains the gold standard for detection and management of intracranial hypertension [1, 2, 10–14, 22, 23]. However, ALF patients, by definition are coagulopathic, and at risk for life-threatening intracranial hemorrhage following monitor insertion [1, 24, 25]. Several prior studies have reported a high incidence of intracranial hemorrhage with IICPM [1, 11, 16, 22]. Most patients in older studies of IICPM in ALF were, however, monitored with subdural/epidural monitors [11, 12, 16, 22, 25–29], Some studies have included patients monitored with external ventricular drains (EVDs) [11]. The ALF Study Group (ALFSG) recommends against the use of EVDs because of the high risk for hemorrhage [1]. Epidural/subdural bolts are rarely used today because of concerns about technical accuracy and overestimation of ICP [29, 30]. Also in these earlier studies, recombinant Factor VIIa (rFVIIa), which is more effective than fresh frozen plasma (FFP) as a reversal agent [1, 14, 31, 32], was not consistently utilized prior to insertion. Intraparenchymal monitors cause less tissue disruption and are associated with a lower risk of hemorrhage compared to EVDs [10, 11, 14, 16, 23]. Some centers have described a relatively low risk of complications with the consistent use of intraparenchymal monitors following administration of rFVIIa [10, 14, 23].

There is a need for more data on the safety and clinical value of IICPM using contemporary devices and reversal agents. The objective of our study was to evaluate the feasibility, safety and impact on clinical management of protocol-driven use of IICPM in a consecutive series of patients with ALF managed at a large liver referral center.

## Methods

In 2011 our institution put in place a protocol for the use of IICPM in ALF. Protocol components included written clinical criteria for invasive monitoring and standardizing the use of agents to reverse coagulopathy prior to insertion. In view of the potential risk associated with the intervention, we created a database to prospectively track complications associated with IICPM use, the frequency with which intracranial hypertension was detected, changes in management following detection of intracranial hypertension and clinical outcomes.

Approval was obtained from the University of Michigan Institutional Review Board (HUM00052556) in 2011 for a quality control database to monitor the complications associated with, and the utility of, invasive monitoring in patients with ALF. Subsequent approval was obtained for this study, to use the database to analyze the outcomes of patients with ALF who underwent IICPM between June 2011 and October 2016 (HUM00120468).

### Outcomes and variables of interest

The primary outcome of interest was the occurrence of IICPM-related intracranial hemorrhage. Additional outcomes studied were the incidence of sustained intracranial hypertension defined as ICP >20 mmHg for >10 mins in a 1-hour period with the patient at rest, therapeutic intensity directed at management of ICP as documented by a therapeutic intensity level (TIL) basic score [33], incidence of all arterial and venous thromboembolic events, incidence of any other complications attributable to IICPM including infection, mortality (all-cause mortality and death from intracranial hypertension), functional status at discharge and functional status at 6 months. The Glasgow Outcome Scale (GOS) [34] was documented at the time of discharge and the 6-month GOS was estimated by chart review of clinic visits following discharge. A GOS of 4 or 5 (able to perform all necessary activities of daily living) was considered to represent a good functional outcome, while GOS 1–3 was judged to be a poor functional outcome.

### Acute liver failure IICPM protocol

All critically ill patients older than 12 years of age, who had ALF and were initially admitted to the Critical Care Medicine Unit (CCMU) of the University of Michigan and evaluated for ICP monitoring, were entered into the database. The criteria subsequently outlined were used to determine eligibility for IICPM.

### Inclusion criteria

1. ALF defined as the onset of hepatic encephalopathy and coagulopathy (i.e. international normalized ratio (INR) >1.5) within 26 weeks of illness onset in a patient without pre-existing liver disease.
2. West-Haven grade-4 hepatic encephalopathy (unresponsive to verbal or noxious stimuli) and Glasgow Coma Scale (GCS) <9 while off sedation for at least 2 hours [3].

Where sedation was deemed necessary, propofol was the agent of choice (with fentanyl as necessary for analgesia and comfort), in view of its short half-life and the consequent ability to obtain a neurological exam when necessary, and a potentially beneficial reduction in cerebral metabolic demand and blood flow [1]. Since propofol was used as the agent of choice, a 2-hour period of sedation interruption was thought to be appropriate, to balance the need for an accurate neurological assessment against the risks of worsening ICP through increasing cerebral metabolic demand. Patients were monitored closely during the 2-hour period of sedation interruption, with particular attention to the pupillary exam, for any signs of decline attributable to intracranial hypertension. While patients with grade-3 encephalopathy may also develop cerebral edema, we included only patients with grade-4 encephalopathy, to restrict the risk of the intervention to the patients at greatest risk of death from intracranial hypertension [35].

### Exclusion criteria

1. No reasonable likelihood of recovery following either medical or surgical intervention, as determined by the gastrointestinal/liver consultant.
2. Complete absence of signs of brainstem function off sedation.
3. Contraindications to rFVIIa or other blood products required for the reversal of coagulopathy.
4. Inability to attain a platelet count >50,000/cm^3^ or fibrinogen >100 mg/dL despite platelet and/or crypoprecipitate transfusion.

Since it is well-established that patients with ALF may demonstrate spontaneous recovery without transplant [1, 17], eligibility for LT was not a requirement for use of IICPM. The purpose of IICPM was to direct clinical management of cerebral edema.

### Reversal of coagulopathy

The protocol utilized for management of coagulopathy is shown in Table 1.

**Table 1:** Protocol for reversal of coagulopathy prior to and following insertion of ICP monitor

Coagulopathy reversal protocol
I. Coagulation parameters to be achieved prior to procedure:
a. Transfusion of pooled platelets to goal platelet count >50,000/cm^3^. b. Transfusion of cryoprecipitate to goal fibrinogen level >100 mg/dL.
II. Immediately before start of procedure:
a. Single dose of desmopressin 0.3mcg/kg IV administered prior to ICP monitor placement. b. Recombinant Factor VIIa 80mcg/kg IV over 2 to 5 minutes. *Following administration of rFVIIa, ICP monitor insertion was performed without repeat laboratory testing to confirm a decrease in the INR*. c. Placement of the ICP monitor within 60 minutes of administration of the rFVIIa dose, clipping and skin preparation to begin within 15–30 minutes of administration of rFVIIa. d. FFP 10 cc/kg infused at the time of monitor insertion regardless of INR
III. Post-procedure:
Repeat laboratory evaluation every 6 hours and correction of coagulopathy, if safe and feasible, to the following goal parameters for a period of 24 hours following placement of the ICP monitor - a. Platelet count >50,000/cm^3^ b. Fibrinogen >100 mg/dL c. INR ≤1.5 Maintenance of these goals for 24 hours could be omitted if the patient was thought to be at high risk of harm from blood product transfusions

*FFP*- Fresh Frozen Plasma, *ICP*- Intracranial Pressure, *INR*- International Normalized Ratio

### Imaging requirements

A non-contrast computed tomography (CT) scan of the brain demonstrating the absence of intracranial hemorrhage was required within a 24-hour period prior to insertion of the ICP monitor. Repeat CT brain imaging was performed within 24 hours of insertion but could be deferred for severe patient instability. All pertinent imaging findings were entered into the database, with particular attention to the presence of hemorrhage, midline shift, unilateral/bilateral sulcal effacement and basal cisternal effacement.

### Insertion and removal of the ICP monitor

Once eligibility was confirmed, informed consent for the use of IICPM was obtained from the appropriate surrogate. Intraparenchymal monitors (Codman™ Microsensor, Codman & Shurtleff Inc, Raynham, MA, USA) were used in all cases. Insertion was performed at the bedside by neurosurgery house-staff. Removal of the ICP monitor was performed after ICP was <20 mmHg for >24 hours, at least 24 hours following LT or rewarming to >37 °C in patients treated with hypothermia. Goal coagulation parameters of INR <1.5, platelet count >50,000/cm^3^ and fibrinogen >100 mg/dL were required prior to removal of the monitor. All patients received prophylactic antibiotic coverage for the duration of ICP monitor use.

### Management of intracranial hemorrhage

In the event of post-procedure intracranial hemorrhage, fresh frozen plasma, cryoprecipitate and platelets were transfused as required to maintain the INR <1.5, platelet count >50,000/cm^3^ and fibrinogen >100 mg/dL. Attempts to reverse coagulopathy were continued until consecutive CT scans at least 24 hours apart demonstrated stability in the size of the hematoma.

### Intracranial pressure - monitoring and management

Any sustained elevation in ICP, defined as ICP >20 mmHg that lasted for >10 mins during a 1-hour period with the patient at rest was recorded in the database. All measures used to control ICP were recorded. A TIL basic score, as originally described in the management of traumatic brain injury (TBI), was estimated based on the treatment measures recorded (Table 2) [33]. All patients received TIL-1 care (basic ICU care), therapy was subsequently escalated through TIL levels to maintain ICP <20–25 mmHg and cerebral perfusion pressure >50–60 mmHg. The first line of therapy for raised ICP despite use of TIL-1 measures was osmotherapy, with intermittent bolus dosing (of mannitol or 23.4% NaCl) or a continuous infusion of 3% NaCl. Raised ICP refractory to osmotherapy was treated with hypothermia to 32–34 °C using the Arctic Sun® external hydrogel device and/or barbiturate coma. Hypothermia was not utilized as a prophylactic or neuroprotective measure, rather as a treatment for established intracranial hypertension refractory to other measures [36–38].

**Table 2:** Therapeutic intensity level (TIL) - basic: a standardized scale of therapeutic intensity directed at control of intracranial pressure, based on the highest attained level for the duration of the patient’s ICU stay

TIL-0: no specific ICP-directed therapy
TIL-1 - basic ICU care
- Sedation for ventilator/endotracheal tube tolerance - Volume/vasopressors for non-CNS cause (e.g. sepsis, myocardial injury) - Head-up positioning (ventilator bundle) - Normocapnia (PaCO2 ≥ 40 mmHg)
TIL-2 - mild
- Higher levels of sedation - Vasopressors/volume for CPP support - Low dose osmotic therapy - Mild hypocapnia (PaCO2 4.6–5.3 kPa; 35–40 mmHg) - CSF drainage <120 ml/day (<5 ml/hour)
TIL-3 - moderate
- Higher doses of osmotic therapy - Moderate hypocapnia (PaCO2 4.0–4.5 kPa; 30–35 mmHg) - Mild hypothermia (>35 ^o^C) - CSF drainage ≥120 ml/day (>5 ml/hour)
TIL-4 - extreme
- Profound hypocapnia (PaCO2 < 4.0 kPa; < 30 mmHg) - Hypothermia <35 ^o^C - Metabolic suppression for control of ICP - Surgery for refractory ICP (decompression, lobectomy)

*CNS*- Central Nervous System, *CPP*- Cerebral Perfusion Pressure, *CSF*- Cerebrospinal Fluid, *ICP*- Intracranial Pressure, *ICU*- Intensive Care Unit, *PaCO2*- Partial pressure of carbon dioxide

### Statistical analysis

A proportion and percentage was described for categorical variables. Mean and standard deviation or median with interquartile range (IQR) was used as appropriate to describe continuous variables. Associations of categorical variables with dichotomous outcomes of interest were evaluated using the chi square or Fisher exact test as appropriate. Associations of continuous variables with dichotomous outcomes of interest were evaluated using the Mann-Whitney U test. Multivariate analysis was performed using a logistic regression model, with occurrence of sustained ICP elevation as the response variable and the following variables, selected on the basis of the existing evidence or biological plausibility, as the explanatory variables: age, gender, GCS, INR, temperature, mean arterial pressure, sodium level, hematocrit, white blood cell count, pH, pO2/FiO2 ratio, ammonia level, occurrence of acute renal failure, sulcal effacement on CT and basal cisternal effacement on CT [7, 17]. All statistical analysis was performed using IBM® SPSS® version 24.

## Results

Thirty-seven consecutive admissions for ALF between July 2011 and October 2016 were evaluated for use of IICPM. Thirteen (35%) patients subsequently did not undergo IICPM. The most common reason for exclusion was an improved neurological examination finding of grade 2–3 encephalopathy when left off sedation, in seven patients (54%). No patient developed clinical decline or new pupillary asymmetry during the period of sedation interruption. Other reasons were the absence of all brainstem function in two patients (15%), rapidly worsening multi-organ failure leading to death within <24 hours in 2 patients (15%) and surrogate refusal of consent in 2 patients (15%). Therefore, a total of 24 patients (65%) underwent IICPM. The median number of days from symptom onset to ICP monitor placement was 5 (IQR 2–10). The distribution of baseline variables and outcomes in patients who did and did not undergo IICPM is depicted in Table 3. Tables 4 and 5 depict the clinical details and outcomes of the 24 patients who underwent IICPM, in survivors of the inpatient admission and deceased patients, respectively.

**Table 3:** Distribution of baseline variables and outcomes among patients with and without use of protocol based IICPM

Variable/outcome	IICPM performed (N = 24)	IICPM not performed (N = 13)	*P* value
Distribution of baseline variables			
Age in years, median (IQR)	39 (23)	44 (24)	0.32
Gender, female (%)	16 (67%)	8 (62%)	0.76
Days from symptom onset to admission, median (IQR)	3 (6)	1 (2.5)	0.22
GCS at time of assessment for ICPM, median (IQR)	3 (2)	9 (4.5)	*0.002*
West Haven grade at time of assessment for ICPM, median (IQR)	4 (0)	3 (2)	*0.019*
Etiology, acetaminophen toxicity (%)	14 (58%)	9 (69%)	0.72
Ammonia in mcg/dL, median (IQR)	119 (86)	86 (88.5)	0.14
Sodium in mmol/L, median (IQR)	142 (12)	140 (8)	0.95
pH, median (IQR)	7.39 (0.20)	7.38 (0.34)	0.56
INR prior to monitor placement, median (IQR)	3 (2)	2.8 (3)	0.79
Platelet count in 10^3^ per microliter, median (IQR)	115 (130)	83 (93)	0.65
Fibrinogen mg/dL, median (IQR)	175 (76)	203 (167)	0.46
Acute renal failure (%)	19 (79%)	9 (69%)	0.69
APACHE 2 score, median (IQR)	28 (10)	25 (9)	0.07
Listed for LT (%)	6 (25%)	1 (8%)	0.38
Liver transplantation performed (%)	4 (17%)	0 (0%)	0.28
Diffuse sulcal effacement on CT (%) at time of initial assessment	6 (25%)	2 (15%)	0.69
Basal cistern effacement on CT (%) at time of initial assessment	2 (8%)	2 (15%)	0.60
Diffuse sulcal effacement on CT (%) at any time	14 (58%)	3 (23%)	0.08
Basal cistern effacement on CT (%) at any time	5 (21%)	2 (15%)	1.00
Distribution of outcomes			
Intracranial pressure (mm Hg), first recorded following insertion, median (IQR)	13 (14)	N/A	
Intracranial pressure (mmHg), maximum recorded, median (IQR)	32 (28)	N/A	N/A
Intracranial hypertension, sustained on ICPM (%)	13 (54%)	N/A	N/A
Therapeutic intensity level, basic score, median (IQR)	2 (3)	1 (0.5)	*0.002*
Intracranial hemorrhage (%)	1 (4%)	0 (0%)	1.00
Thromboembolic events (%)	0 (0%)	0 (0%)	1.00
Brain death (%)	1 (4%)	2 (15%)	0.28
Death from intracranial hypertension (%)	2 (8%)	2 (15%)	0.60
Dead or severely disabled (GOS <4) at discharge (%)	19 (79%)	11 (85%)	1.00
Dead at discharge (%)	12 (50%)	6 (46%)	1.00
Alive but severely disabled/vegetative at discharge (%)	7 (29%)	5 (38%)	0.72
Dead or severely disabled (GOS <4) at 6 months (%)	14/18 (78%)	6/8 (75%)	1.00
Dead at 6 months (%)	12/18 (67%)	6/8 (75%)	1.00
-Alive but severely disabled/vegetative at 6 months (%)	2/18 (11%)	0/8 (0%)	1.00

*IICPM* invasive intracranial pressure monitoring, *GCS* Glasgow Coma Score, *APACHE* Acute Physiology and Chronic Health Evaluation, *LT* liver transplantation, *CT* computed tomography, *GOS* Glasgow Outcome Scale, *N/A* Not Applicable

*p* values in italic font signify statistical significance (*p* <0.05)

**Table 4:** Patients who underwent IICPM and were alive at discharge

Patient number	Etiology	Liver transplant (yes/no)	Sustained ICP elevation (yes/no)	TIL (basic)	GOS at discharge	GOS at 6 months
1	Epstein Barr virus	Yes	Yes	2	4	5
2	Idiopathic	Yes	No	1	3	5
3	Hepatitis B	Yes	Yes	2	3	3
4	Acetaminophen	No	No	1	4	N/A
5	Acetaminophen	No	No	1	3	N/A
6	Ischemic	No	Yes	2	3	3
7	Highly active anti-retroviral therapy	No	No	1	3	N/A
8	Acetaminophen	No	Yes	2	4	N/A
9	Acetaminophen	No	No	1	4	N/A
10	Acetaminophen	No	Yes	4	3	5
11	Acetaminophen	No	Yes	2	4	N/A
12^a^	Acetaminophen	No	No	1	3	5

Descriptive table of patients who underwent invasive intracranial pressure monitoring (IICPM), and were alive at discharge, with individual functional outcomes *ICP* intracranial pressure, *GOS* Glasgow Outcome Scale, *TIL* therapeutic intensity level, *N/A* Not Applicable

^a^The only patient to suffer a complication of IICPM - an asymptomatic 6-mm subdural hematoma

**Table 5:** Patients who underwent IICPM and died prior to discharge

Patient number	Etiology	LT (yes/no)	Sustained ICP elevation (yes/no)	TIL (basic)	ICP control achieved?	Cause of death
1	Hepatitis B	Yes	Yes	2	Yes	Cardiac arrest from blood loss during transplantation
2	Idiopathic	No	No	1	Yes	Severe sepsis, multi-organ failure, absence of liver recovery, withdrawal life support
3	Phenytoin	No	No	1	Yes	Absence of liver recovery, progressive multi-organ failure and withdrawal life support
4	Acetaminophen	No	No	1	Yes	Absence of spontaneous liver recovery, progressive multi-organ failure and withdrawal life support
5	Acetaminophen	No	Yes	4	Yes	Absence of liver recovery, progressive multi-organ failure and withdrawal life support
6	Acetaminophen	No	No	1	Yes	Absence of liver recovery, progressive multi-organ failure and withdrawal life support
7	Acetaminophen	No	Yes	4	No	Progression to brain death
8	Acetaminophen	No	Yes	4	Yes	Absence of liver recovery, progressive multi-organ failure and withdrawal life support
9	Acetaminophen	No	Yes	2	Yes	ICP controlled but multiple infarcts including cerebellar stroke on imaging thought to be from prior high ICP and hypoperfusion. Withdrawal of life support for likely devastating neurological injury
10	Idiopathic	No	Yes	4	Yes	Absence of liver recovery, progressive multi-organ failure and withdrawal life support
11	Acetaminophen	No	No	1	Yes	Absence of liver recovery, progressive multi-organ failure and withdrawal life support
12	Hepatitis A, EBV	No	Yes	3	Yes	Severe sepsis and multi-organ failure despite ICP control and laboratory evidence of liver recovery

Descriptive table of patients who underwent invasive intracranial pressure (ICP) monitoring (IICPM), and were dead at discharge, with individual cause of death. *LT* liver transplantation, *TIL* therapeutic intensity level, *EBV* Epstein-Barr virus

### Protocol implementation

All elements of the coagulopathy reversal protocol (Table 1), including platelet and fibrinogen goals, were successfully implemented prior to monitor insertion in all 24 patients. Insertion occurred within 60 minutes of the rFVIIa dose in all cases. No patient received repeat doses of rFVIIa. Administration of blood products for reversal of coagulopathy for the 24-hour period following ICPM insertion was deferred in five patients (22%) because of the perceived risk of harm. No patient had intracranial hemorrhage on the pre-procedure CT scan, available for all patients. At least one non-contrast CT brain scan was performed in the 24-hour period following monitor insertion in all patients.

### Complications

Intracranial hemorrhage occurred in a single patient (1/24, 4%), an asymptomatic 5-mm right-sided subdural hematoma at the insertion site with mild mass effect on the underlying frontal lobe and no midline shift. Reversal of coagulopathy was performed (successfully) for a 72-hour period in this patient and serial CT scans over 72 hours showed no further expansion. There were no episodes of sustained ICP elevation. The patient demonstrated spontaneous recovery without LT, was subsequently extubated and underwent removal of the ICP monitor 8 days following insertion. She was neurologically intact at the time of discharge, with a GOS of 5 at 6 months. No patient had a documented thromboembolic complication and no patient suffered an infection related to IICPM use.

### Sustained intracranial hypertension

Of 24 patients who underwent ICP monitoring, 13 (54%) had at least one sustained episode of intracranial hypertension (Tables 3, 4 and 5); 5/24 (21%) had sustained ICP elevation recorded immediately following insertion. The median duration from onset of grade-4 encephalopathy to sustained ICP elevation in the 13 patients who developed sustained ICP elevation was 6 hours (IQR 24 hours). The median initial ICP recorded was 13 mmHg (IQR 14), while the median maximum recorded ICP was 32 mmHg (IQR 28). Of note, there was no significant difference in the lowest GCS between patients who developed sustained intracranial hypertension and monitored patients whose ICP remained normal (the median lowest GCS was 3 T in both groups, *p* = 1.00). There was no significant association between the occurrence of sustained intracranial hypertension and diffuse sulcal effacement on initial CT (*p* = 0.65), diffuse sulcal effacement at any time (*p* = 1.00), basal cisternal effacement on initial CT (*p* = 1.00) or basal cisternal effacement at any time (*p* = 0.33). No single variable attained statistical significance for prediction of sustained ICP elevation in the logistic regression model; however, the limited number of patients in whom this event occurred (n = 13 of 24) greatly limited the number of variables that could be included and the predictive value of the model.

### Therapeutic intensity

All 11 IICPM patients who never developed sustained ICP elevation had a TIL-basic score of 1 for the duration of their ICU stay. Therapeutic intensity attained in 13 patients with sustained ICP elevation was TIL-2 in seven patients (54%), TIL-3 in one patient (8%) and TIL-4 in five patients (38%). While 4/5 patients requiring TIL-4 (extreme measures) achieved effective ICP control, only one survived, a 15-year-old with acetaminophen toxicity treated with hypothermia and pentobarbital, who had spontaneous recovery of liver function. Among patients who attained TIL-4, two were treated with a pentobarbital infusion alone, one with hypothermia to 32–34 °C alone, and two with both pentobarbital and hypothermia to 32–34 °C. Hypothermia was used to control established, refractory, intracranial hypertension in all three cases, rather than as a prophylactic or neuroprotective measure. No patient underwent decompressive craniectomy.

### Liver transplantation

Of 37 patients evaluated, 30 (81%) were deemed to have contraindications to LT. These included active alcohol or substance abuse in 8/30 patients (27%), clinical improvement and/or laboratory signs of hepatic recovery in 8 (27%), rapidly developing multi-organ failure with suspected sepsis in 4 (13%), multiple prior suicide attempts in 3 (10%), acute myocardial infarction with hemodynamic instability in 2 (7%), nonadherence to medical therapy in 2 (7%), rapid progression to brain death in 2 (7%) and cystic fibrosis in 1 (3%). Of seven patients initially listed for LT, two developed rapid clinical decline and died of multi-organ failure prior to transplant while one was taken off the transplant list for evidence of active substance abuse. Four patients underwent LT, all four underwent IICPM. Two of these patients demonstrated an excellent outcome, with good functional outcome at 6-month follow up. A third patient survived but with significant cognitive impairment. At 6 months she was able to perform basic activities of daily living but unable to live independently (GOS 3, severe disability). The fourth patient died during transplantation following uncontrolled bleeding and intra-operative elevation of ICP. Sustained intracranial hypertension was observed in two of the three surviving patients who underwent LT. One of these patients developed elevated ICP exclusively during surgery, managed with elevation of the CPP alone (TIL-2), and the other patient had both preoperative and intra-operative sustained elevation in ICP managed with mannitol (TIL-2).

### ICP monitor removal

Of 12 patients who died following IICPM placement, 11 had the monitor present at the time of death while one had the monitor removed following 48 hours of normal ICP. Among 13 patients who survived, removal of IICPM was performed a median of 4.5 (IQR 4) days following placement. Only one of these patients required transfusion of FFP at the time of removal, the others had regained normal coagulation parameters (without correction) at the time of monitor removal.

### Mortality

The individual outcomes of the 24 patients who underwent IICPM are shown in Table 4 (patients who were alive at discharge) and Table 5 (patients who died prior to discharge). Overall, 18 of 37 patients (49%) died during the inpatient admission. Notably only 4/18 (22%) of all deaths and 2/12 (17%) deaths among IICPM patients were a consequence of intracranial hypertension.

### Functional outcome

Of 19 patients with ALF who survived the inpatient admission, 7 (37%) were judged capable of independent living at the time of discharge (GOS 4), while 12 (63%) were ambulatory but still required some assistance at discharge (GOS 3). Six-month follow up was available from clinical records for eight survivors; six were asymptomatic (GOS 5) at 6 months while two continued to require some assistance with daily living (GOS 3).

## Discussion

Our study demonstrates that protocol-based IICPM use in patients with ALF is both feasible and safe. Only one patient (4%) suffered hemorrhage, which was a 5-mm asymptomatic subdural hemorrhage in a patient who had a good outcome. Other notable findings are that over half of monitored patients developed at least one episode of sustained intracranial hypertension requiring TIL >1, and about one in five developed refractory intracranial hypertension requiring extreme measures for ICP control (TIL-4). Our study also demonstrates that excellent outcomes are possible, including inpatients not deemed eligible for LT. This is illustrated by the 15-year-old patient deemed ineligible for LT who required TIL-4 care (barbiturate coma and hypothermia) before demonstrating spontaneous liver recovery and eventual excellent outcome (GOS 5 at 6 months).

Table 6 lists the studies that have described the use of IICPM in ALF [10–14, 16, 21–23, 26–29, 37]. Our study is distinct for several reasons. All data points were prospectively collected to monitor the safety and quality of a potentially risky procedure. While larger multi-center studies have reported the use of a mix of devices (including subdural/epidural catheters and EVDs) and reversal strategies [11, 16, 22], this is the largest reported series from a single center with protocol-driven use of intraparenchymal catheters and rFVIIa in all patients. Most important, unlike prior studies, every patient who underwent IICPM received follow-up imaging, thereby providing a reliable estimate of the rate of intracranial hemorrhage. The low rate of hemorrhage in our study, with no symptomatic bleeds, may, therefore, be particularly noteworthy.

**Table 6:** Published studies of invasive intracranial pressure monitoring in ALF

Year of publication	Author	Single or multi-center	Number of patients	Type of invasive monitor used	Coagulopathy reversal strategy	Consistent use of a single protocol?	Post-procedure imaging required in all patients?	Incidence of intracranial hemorrhage	Incidence of elevated ICP
1980	Hanid et al.	Single-center	10	Subdural 100%	FFP 2 units during procedure	Yes	No	14%	100%
1982	Canalese et al.	Single- center clinical trial of mannitol and dexamethasone	17	Subdural and epidural	FFP during procedure	Yes	No	None reported	82%
1989	Forbes et al.	Single-center clinical trial of thiopental in ALF	13	Epidural 100%	Not specified	N/A	No	None reported	100%
1991	Munoz et al.	Single-center	14	Epidural 100%	FFP and plasma exchange to goal prothrombin time <16 seconds	Yes	No	None reported	80%
1992	Lidofsky et al.	Single- center	23 (20 adults, 3 children)	Subdural 91%; epidural 9%	FFP to goal prothrombin time within 3–5 seconds of control; fibrinogen goal 100 mg/dL; platelet transfusion to goal 75,000/μL	Yes	No	22% (symptomatic 17%)	43%
1993	Keays et al.	Single-center	36	Epidural 100%	None	N/A	No	3% (symptomatic 0%)	26%
1993	Blei et al.	Multi-center	262	Epidural 61%; subdural 30%; intraparenchymal 9%	Not specified	No	No	Overall 8% (fatal 4%); epidural 3% (fatal 1%); subdural 18% (fatal 5%); intraparenchymal 13% (fatal 4%)	Not evaluated
2005	Vaquero et al.	Multi-center (ALFSG)	58	Subdural 64%; intraparenchymal 21%; epidural 16%	FFP 91%; platelets 31%; rFVIIa 26%; cryoprecipitate 19%; plasmapheresis 5%	No	No	Overall 10% (symptomatic 5%); subdural 11% (symptomatic 8%); epidural 11% (symptomatic 11%), intraparenchymal 8% (symptomatic 0%)	Not reported
2008	Raschke et al.	Single-center	22	Intraparenchymal 100%	rFVIIa plus FFP to goal prothrombin time <16 seconds; platelet transfusion to goal >100,000/μL; fibrinogen goal 100 mg/dL; desmopressin 30 μg/kg IV × 1	Yes	No	3/17 (18%) overall	95%
2010	Le et al.	Single- center	11	Intraparenchymal 100%	rFVIIa average dose 3 mg (36.7 μg/kg), monitor then placed within 2 hours without rechecking INR	Yes	Yes	None	None
2012	Kamat et al.	Single-center	14 children	Intraparenchymal 100%	rFVIIa plus FFP within 30 mins prior to procedure to achieve INR ≤1.5; vitamin K	Yes	Not reported	Symptomatic 7%	Not reported
2014	Karvellas et al.	Multi-center (ALFSG)	140	Subdural 27%; intraparenchymal 24%; epidural 23%; lumbar 17%; external ventricular drain 9%;	FFP 84%; platelet transfusion 43%; rFVIIa 2%	No	No	Symptomatic 7%	51%
2016	Maloney et al.	Single-center	20	Intraparenchymal 65%; epidural 35%	FFP and rFVIIa to goal INR ≤1.5; platelet transfusion to goal 50,000/μL	Not reported	No	Overall 15% reported. intraparenchymal - 2 of 10 (20%) with imaging available (both fatal); epidural 1 of 3 (33%) - with imaging available (asymptomatic)	70%
2016	Bernal et al.	Multi-center clinical trial of hypothermia in ALF	43	Intraparenchymal 100%	Not reported	Not reported	No	None - one remote hemorrhage in the temporal lobe	44%
Current study	Rajajee et al.	Single-center	24	Intraparenchymal 100%	See Table 1	Yes	Yes	4% (symptomatic 0%)	54%

List of studies of invasive intracranial pressure monitoring in acute liver failure (ALF). *ALFSG* Acute Liver Failure Study Group, *FFP* fresh frozen plasma, *rFVIIa* Recombinant factor VIIa, *IV* intravenous, *INR* international normalized ratio, *N/A* Not Applicable

We believe that protocol-driven reversal of coagulopathy is essential in this regard. Considering the relatively short 2-hour half-life of rFVIIa, our protocol placed a heavy emphasis on monitor placement within an hour of a dose sufficient to reliably achieve normalization of the INR [1, 31, 39–41], without repeat laboratory testing which may delay placement. The use of rFVIIa may achieve normalization of the INR without reversal of all of the elements of the coagulopathy associated with ALF. Our protocol therefore included a requirement that FFP be transfused at the time of monitor insertion (Table 1) in all cases [1]. A recent meta-analysis did not demonstrate a reduction in transfusion requirements with the use of rFVIIa in the setting of hepatic surgery [42], however, and rFVIIa is not universally accepted as the standard of care for the reversal of coagulopathy prior to invasive procedures in the setting of ALF. [43] In particular, the efficacy of rFVIIa may be degraded by acidosis, with one study suggesting a loss of efficacy of over 90% with a drop in the pH from 7.4 to 7.0 [44]. It is therefore relevant that the median pH in patients who underwent IICPM, and therefore received rFVIIa, was 7.39 in our study - rFVIIa may be less effective, and hemorrhage risk possibly higher, in a patient population with a higher incidence of severe, uncorrected acidosis at the time of the invasive procedure. It is also important to note that the low incidence of procedural hemorrhage in our study may primarily reflect the other elements of coagulopathy reversal that were part of the protocol, such as those directed at platelet count and function (platelet transfusion to goal >50,000/cm^3^ plus use of DDAVP in all cases) and adequate fibrinogen levels (goal >100 mg/dL), rather than the use of rFVIIa. A comprehensive approach to the coagulopathy of ALF may be critical to mitigate the risk of procedural bleeding.

Our study is also unique in providing information on the therapeutic intensity level directed at ICP management, which specifically provides a measure of the impact of IICPM on clinical management. It is important to note that all escalation in therapeutic intensity above TIL-1 in our study occurred exclusively on the basis of the measured ICP, rather than the clinical examination, which was already very poor, or imaging, which was only repeated in the first post-procedure day to look for hemorrhage.

Our tracking of long-term outcomes confirms that patients may survive ALF with excellent outcome despite the occurrence of intracranial hypertension, but some may also be left with long-term cognitive dysfunction from potentially preventable secondary brain injury. Another interesting observation in our study is that, in contrast to earlier studies of ALF [3–5, 45], and consistent with some more recent studies [1, 17, 46], intracranial hypertension was not the primary cause of death - 87% of monitored patients died of progressive multi-organ failure (following effective control of ICP) and only 9% died as a direct consequence of intracranial hypertension. It is possible that the use of IICPM may decrease the frequency of death from intracranial hypertension, while overall survival remains dependent on timely emergency LT or spontaneous recovery of liver function.

The major argument in favor of invasive monitoring is that IICPM frequently reveals the presence of otherwise unsuspected sustained intracranial hypertension requiring therapy. Patients in grade-4 encephalopathy are all deeply comatose with a GCS of 3–4 T, as seen in our study, providing limited opportunity for detection of raised ICP through further neurological decline until cerebral herniation and pupillary asymmetry occurs. In our study, CT findings such as sulcal effacement were poorly predictive of intracranial hypertension, a finding consistent with other studies in patients with ALF [12, 21]. It is also impractical to perform serial imaging with CT in patients with ALF, who frequently develop severe hemodynamic and respiratory instability. The use of IICPM permits both escalation of therapy in patients who do not respond to first line measures (and may progress to cerebral herniation/brain death without more aggressive measures), and withholding of treatment measures with potentially serious side effects in patients whose measured ICP is normal (46% of monitored patients in our study). This includes avoidance of the potential side effects of mannitol (hypovolemia, worsening renal function), hypertonic saline (acidosis, circulatory overload), deep sedation (hypotension, prolongation of mechanical ventilation and ICU length of stay), hypothermia (worsening coagulopathy, infection) and barbiturates (prolonged sedation, hypotension, ileus, pneumonia), among others. The impact of IICPM on clinical management was most evident in the 21% of monitored patients requiring extreme measures such as barbiturate coma or hypothermia (TIL-4), which successfully controlled ICP in 4/5 patients. Less invasive monitoring tools such as jugular venous oximetry and noninvasive tools such as optic nerve ultrasound and transcranial Doppler may have a future role in the assessment of ICP and cerebral perfusion in this population, given the risks and expense associated with IICPM [47–55]. Their ability to accurately detect elevated ICP and direct appropriate changes in management is uncertain, however, and their role in the management of ALF is currently investigational. Our group is currently studying the accuracy of optic nerve ultrasound and transcranial Doppler compared to IICPM for the prediction of intracranial hypertension in ALF.

The primary limitations of our study are a small sample size and its descriptive nature. Our study cannot provide definitive evidence favoring the routine use of IICPM in patients with ALF. Since ALF is an uncommon condition, and IICPM is only performed in a subset of major liver centers, a randomized clinical trial focused on functional outcomes, with an adequate sample size, will likely be challenging to conduct. Clinical trials of monitoring tools are also inherently limited by the fact that outcomes are determined by the actions (or lack thereof) taken in response to the monitoring tool. In the BEST-TRIP clinical trial of IICPM following traumatic brain injury (TBI), statistically significant differences in therapies used to manage ICP may have played a significant role in determining outcomes, beyond simply the use of IICPM [56]. While the ALFSG has pooled information from multiple centers to provide valuable data from large patient samples, retrospective comparisons of outcomes in patients who do and do not receive IICPM in these studies may be limited by potential confounding from selection bias as well as the absence of a common protocol and the consequent heterogeneity in patient selection, devices, reversal strategies and therapies used to control ICP [11, 16, 22]. Illustrating the potential for selection bias, patients who underwent IICPM in our study had greater disease severity, with worse GCS (*p* = 0.002), as a consequence of the protocol eligibility criteria. While the 2-hour period of sedation interruption may have resulted in a transient increase in cerebral metabolic demand, several patients (7 of 37 screened) were in fact found to be at a better neurological grade than initially suspected, following sedation interruption, and, as a consequence, did not undergo monitor placement. None of these patients subsequently died as a result of intracranial hypertension. We therefore believe that sedation interruption was necessary, to restrict the risks of the intervention to as few patients as possible - those with the highest risk of death from elevated ICP.

## Conclusion

Protocol-based use of invasive intracranial pressure monitoring following acute liver failure is feasible, is associated with a low incidence of serious complications and has a significant impact on management.

## Abbreviations

ALF: Acute liver failure
ALFSG: Acute Liver Failure Study Group
APACHE 2: Acute Physiology and Chronic Health Evaluation 2
CCMU: Critical Care Medicine Unit
CO2: Carbon dioxide
CPP: Cerebral perfusion pressure
CT: Computed tomography
EBV: Epstein Barr virus
EVD: External ventricular drain
FFP: Fresh frozen plasma
GCS: Glasgow Coma Scale
GOS: Glasgow Outcome Scale
HAART: Highly active antiretroviral therapy
HELLP: Hemolysis/elevated liver enzymes/low platelet
IBM: International business machines
ICP: Intracranial pressure
ICU: Intensive Care Unit
IICPM: Invasive intracranial pressure monitoring
INR: International normalized ratio
IQR: Interquartile range
LT: Liver transplantation
MA: Massachusetts
NaCl: Sodium chloride
PaCO2: Partial pressure or carbon dioxide
rFVIIa: Recombinant factor VIIa
TBI: Traumatic Brain Injury
TIL: Therapeutic intensity level
